# Amplicon sequencing dataset of soil fungi and associated environmental variables collected in karst and non-karst sites across Yunnan province, southwest China

**DOI:** 10.1016/j.dib.2019.104575

**Published:** 2019-09-27

**Authors:** Kingsly C. Beng, Richard T. Corlett

**Affiliations:** a Center for Integrative Conservation, Xishuangbanna Tropical Botanical Garden, Chinese Academy of Sciences, Menglun, Mengla, Yunnan, 666303, China; b Center of Conservation Biology, Core Botanical Gardens, Chinese Academy of Sciences, Menglun, Mengla, Yunnan, 666303, China

**Keywords:** Metabarcoding, Karst, Soil pH, Saprotrophs, Ectomycorrhizal fungi, Mycology, Limestone, Internal transcribed spacer

## Abstract

Fungi are among the most widely distributed organisms on Earth, performing key roles in nutrient cycling, disease, and the global carbon cycle. However, studies on regional-scale fungal assemblage patterns and the underlying drivers, are scarce. The aim of this research was to determine the relative importance of environmental heterogeneity and spatial distance on the metacommunity structure of soil fungi in Yunnan province, southwest China. This dataset is supplementary to research by [1] and presents 12,843 fungal operational taxonomic unit (OTU) sequences, OTU distribution and abundance across 220 samples, OTU taxonomic and ecological annotations, and environmental characteristics of the sites where the samples were collected. Differences in fungal alpha and beta diversity indices between karst and non-karst soils for the full dataset, six class-level (Agaricomycetes, Dothideomycetes, Sordariomycetes, Leotiomycetes, Tremellomycetes, and Eurotiomycetes) and four functional-level (symbiotrophs, pathotrophs, saprotrophs, and ectomycorrhizal fungi) datasets are presented.

**Table:** Specifications Table

Subject	Environmental Science Ecology
Specific subject area	Fungi internal transcribed spacer 2 (ITS2) metabarcoding
Type of data	Text files (DNA sequences in fasta, OTU-table) Excel Figures Graphs
How data were acquired	We homogenized soil samples by vortexing and extracted genomic DNA from 0.5 g representative subsamples using the TIANamp Soil DNA Kit (TIANGEN Biotech Co.,Ltd, Beijing) according to the manufacturer's instructions. We used Qubit 2.0 to quantify DNA concentration and diluted the DNA of each sample to 20 ng/ul final concentration. We amplified the internal transcribed spacer 2 (ITS2) region of the nuclear ribosomal gene using the primers fITS7/ITS4 [2,3] on an Applied Biosystems 2720 Thermal Cycler (Life Technologies, CA, USA). We prepared sequencing libraries using the TruSeq Nano DNA LT Library Prep Kit, and pooled them in equimolar amounts. We paired-end sequenced (2 × 250 bp) the pooled libraries on Illumina Miseq. We recorded the latitude, longitude and elevation of sample locations using a handheld Global Positioning System (GPS, GARMIN GPSMAP 62s). We measured (i) soil pH in a 1:2.5 mixture of soil to water using a digital pH meter (FE28, Mettler Toledo, USA); (ii) total Carbon (TC) and Nitrogen (TN) using a CN analyzer (Vario MAX CN; Elementar, Germany); (iii) total Phosphorus (TP), Potassium (TK), Calcium (TCa) and Magnesium (TMg) using ICP-AES (iCAP6300, Thermo Fisher Scientific, USA). We downloaded the standard 19 bioclimatic variables (BIO1-BIO19) for each sample location at 30 seconds (∼1 km^2^) spatial resolution from Worldclim version 2 [4] using the R raster package [5].
Data format	Raw Analysed Filtered Visualizations
Parameters for data collection	We selected 44 karst and non-karst forest sites covering a wide range of environmental conditions (elevation, slope, aspect, soil) for soil fungi metabarcoding.
Description of data collection	In each site, we established five 15 m × 15 m inventory plots and collected nine soil cores from each plot.
Data source location	Xishuangbanna Tropical Botanical Garden, Chinese Academy of Sciences Menglun, Mengla, Yunnan, China 21.73–25.42 N, 101.41–102.73 E
Data accessibility	With the article
Related research article	K.C. Beng, R.T. Corlett, Identifying the mechanisms that shape fungal community and metacommunity patterns in Yunnan, China; Fun Ecol; in press [1].

**Table:** 

**Value of the data** • This dataset represents fungal metacommunities from karst and adjacent non-karst forest sites, as well as the environmental characteristics of each metacommunity. Ecological analyses using this data can improve our understanding of how fungal communities are organized along environmental gradients and how these patterns of community assembly relate to underlying ecological processes. • Identifying the mechanisms that underlie fungal distribution and the processes that drive fungal community structure is crucial for predicting how ecosystems will respond to global environmental change. This information is of great importance to researchers interested in plant-soil feedbacks, mycorrhizal interactions, and symbiotic relationships between fungi and animals. • This research mainly focused on soil fungi in the tropics and subtropics. The experimental design and statistical approach used here can be replicated across other groups of organisms (e.g. bacteria, viruses, archaea, and invertebrates), ecosystems (e.g. marine and freshwater) and biomes (e.g. temperate, boreal, and tundra), and the results obtained can be used to compare community responses between and among groups, ecosystems, and biomes. • The raw sequences are available at the National Center for Biotechnology Information (NCBI) Sequence Read Archive (SRA) under accession number SRP158134. Researchers interested in fungal ecology and/or biogeography can further explore this dataset using bioinformatic protocols of their choice or combine this data with their own data. • Due to the incompleteness of fungal reference sequence databases, some OTUs have not been assigned to taxonomic or functional groups. This will hopefully stimulate more people to build up reference sequence databases for fungi. Improving the barcode database needs to be a higher priority in tropical areas.

## 1 Data

This dataset includes raw DNA sequence data obtained through Illumina Miseq paired-end (2 × 250 bp) amplicon sequencing of the ITS2 region of fungi in 220 soil samples distributed across 44 karst and non-karst forest sites. The raw data (DNA sequences in FASTQ format) have been deposited at NCBI SRA database under project accession No. SRP158134. DNA sequences (representing 100% OTUs) from bioinformatics analysis of raw sequence data (denoising, dereplication, clustering, and filtering) are presented in Supplemental file 1. The OTU-by-site matrix (OTU table) with the frequency of each OTU per site, and OTU taxonomic and functional annotations, is presented in Supplemental file 2. A metadata file, including the location and elevation, soil variables measured, bioclimatic variables downloaded, and spatial distances computed for each of the 220 plots sampled, is presented in Supplemental file 3.

Information on how karst and non-karst sites were identified, on sampling design, and on diversity (alpha and beta) estimates between karst and non-karst fungi, is presented in Fig. 1, Fig. 2, Fig. 3, Fig. 4, Fig. 5, Fig. 6, Fig. 7, Fig. 8, Fig. 9, Fig. 10, Fig. 11, Fig. 12, Fig. 13.

**Fig. 1:**
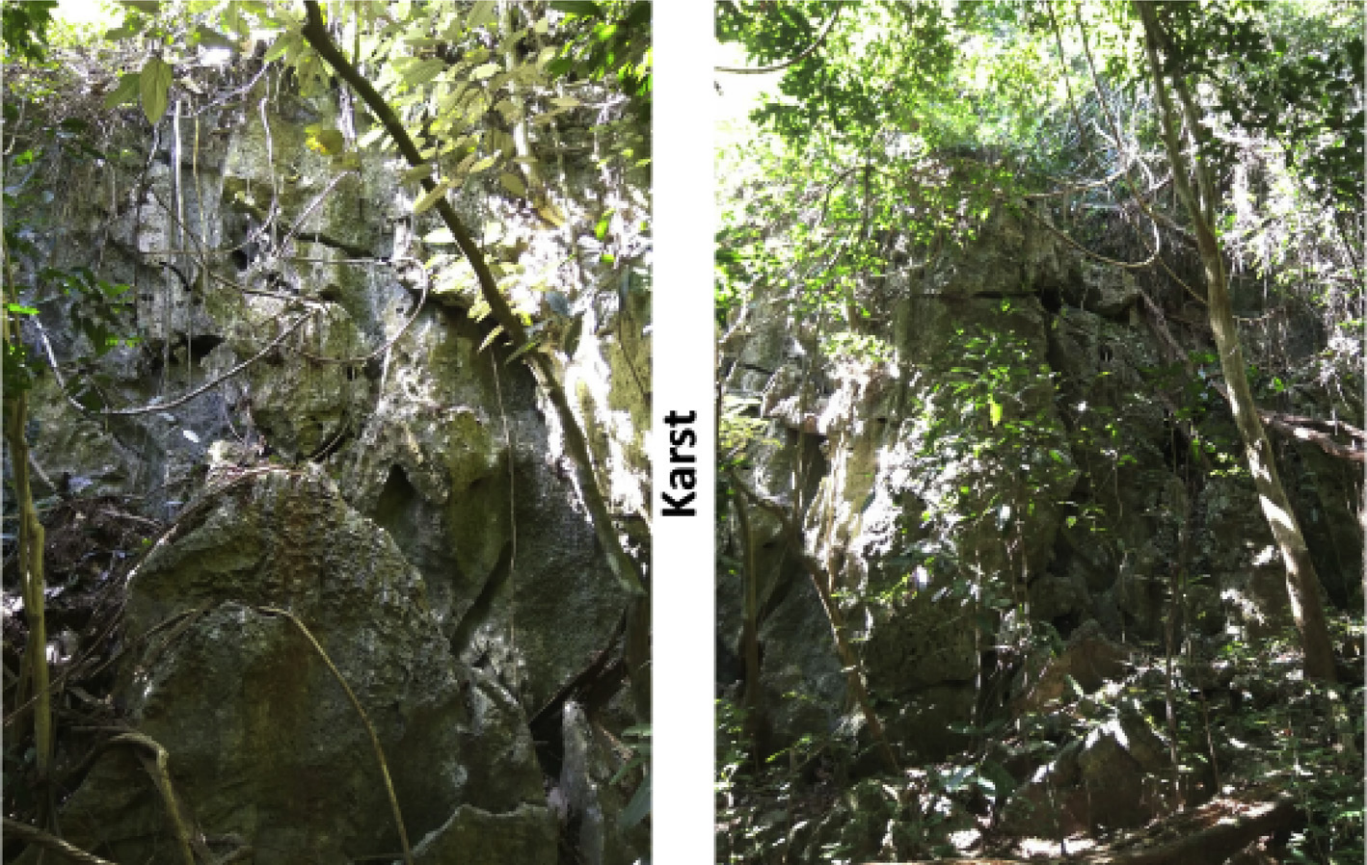
Rock outcrops pictured in two of the karst forest sites in our study. Photo by K. C. Beng.

**Fig. 2:**
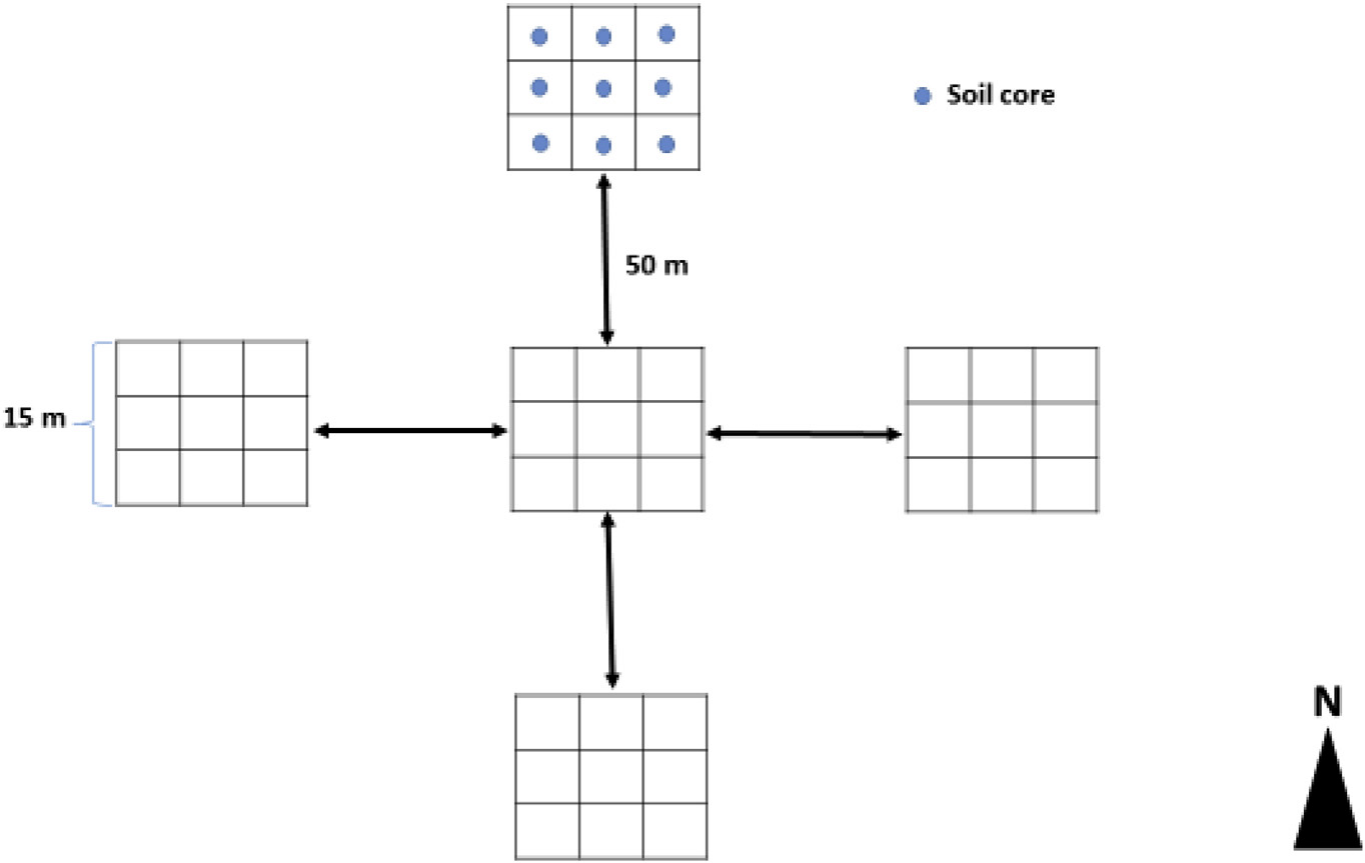
Schematic diagram of sampling design showing the five plots per site, the approximate distance between any two plots and the nine soil cores collected per plot.

**Fig. 3:**
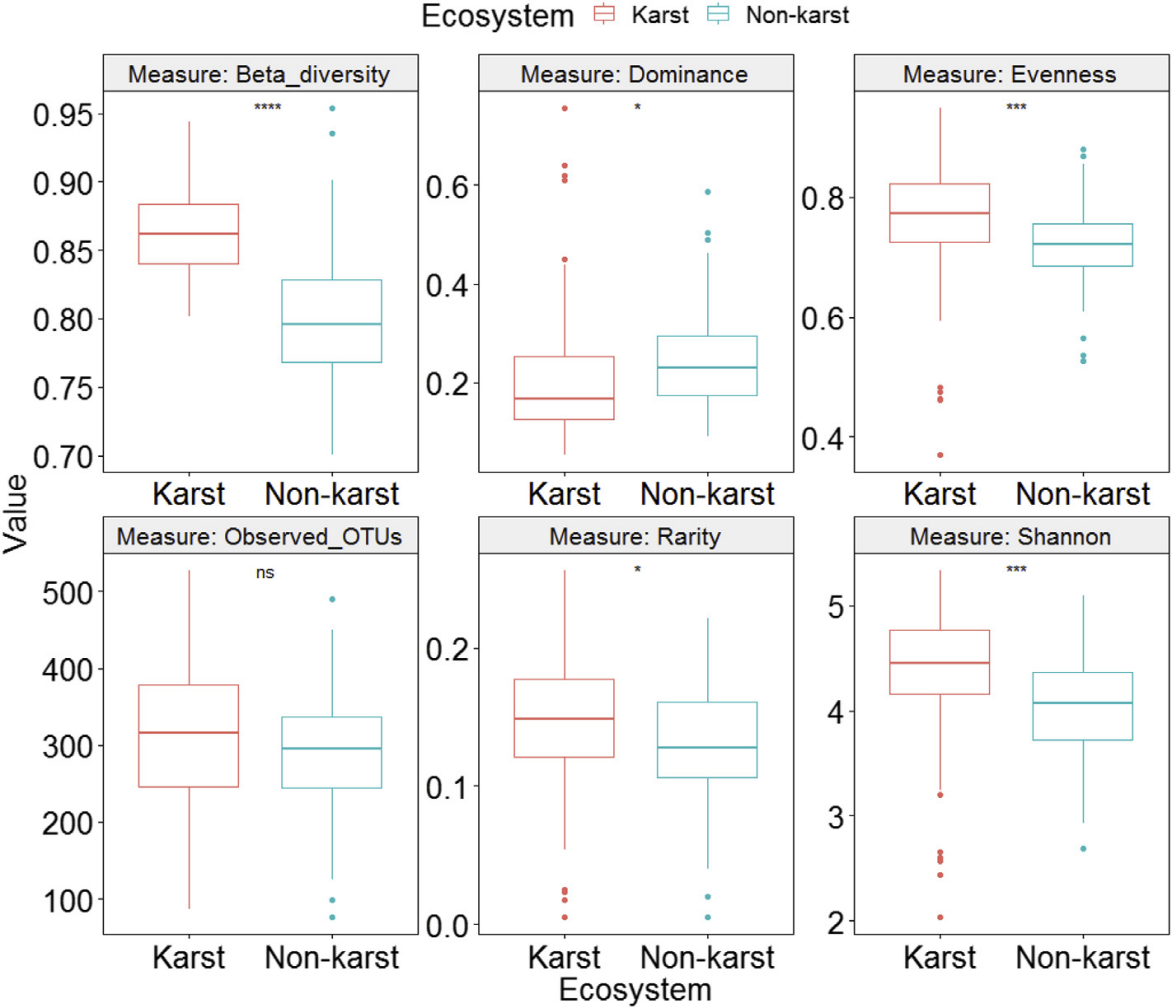
Differences in fungal alpha and beta diversity indices between karst and non-karst soils for the full dataset (Supplemental file 4) after rarefying to equal number of sequences (12,723) per sample.

**Fig. 4:**
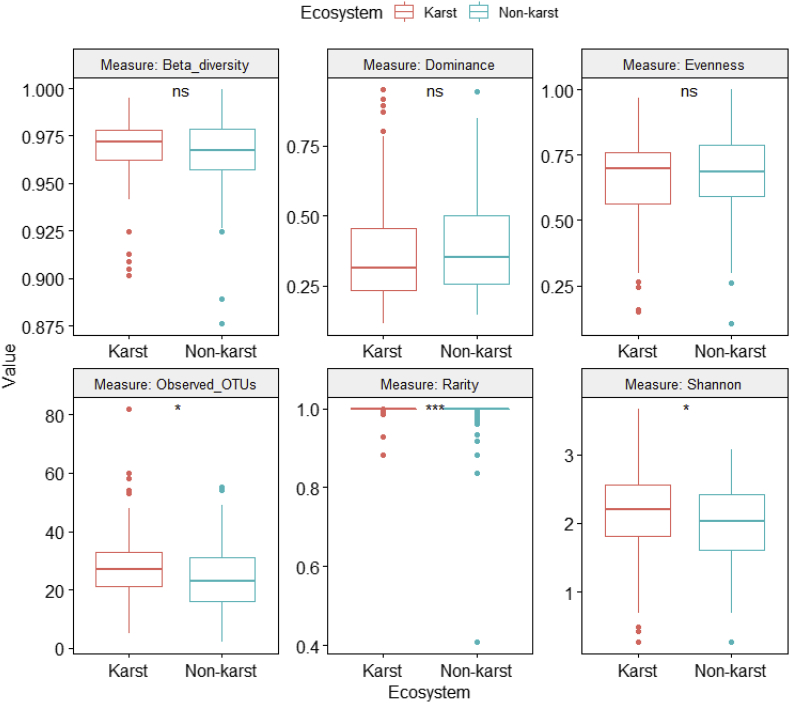
Differences in fungal alpha and beta diversity indices between karst and non-karst soils for the Agaricomycetes dataset (Supplemental file 5).

**Fig. 5:**
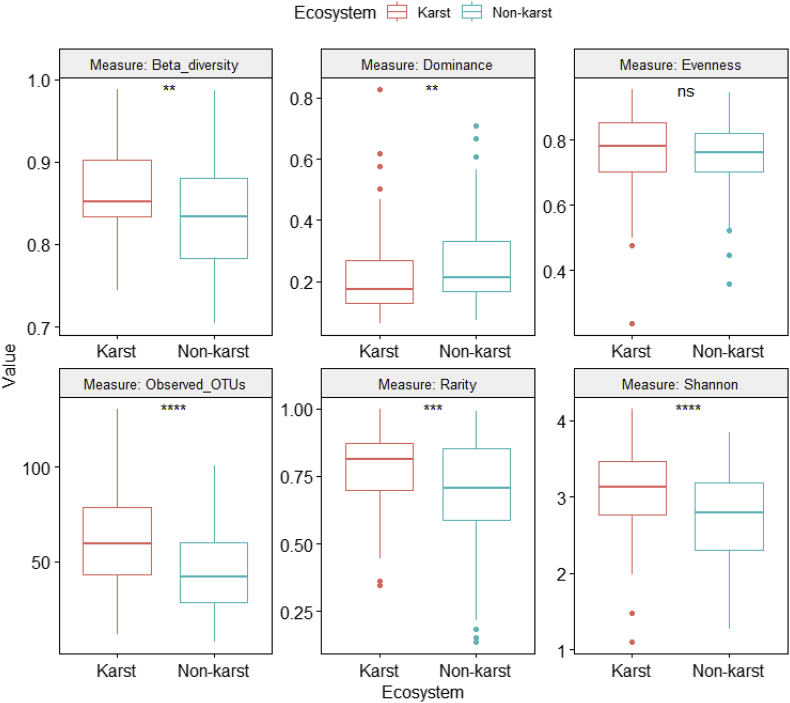
Differences in fungal alpha and beta diversity indices between karst and non-karst soils for the Dothideomycetes dataset (Supplemental file 6).

**Fig. 6:**
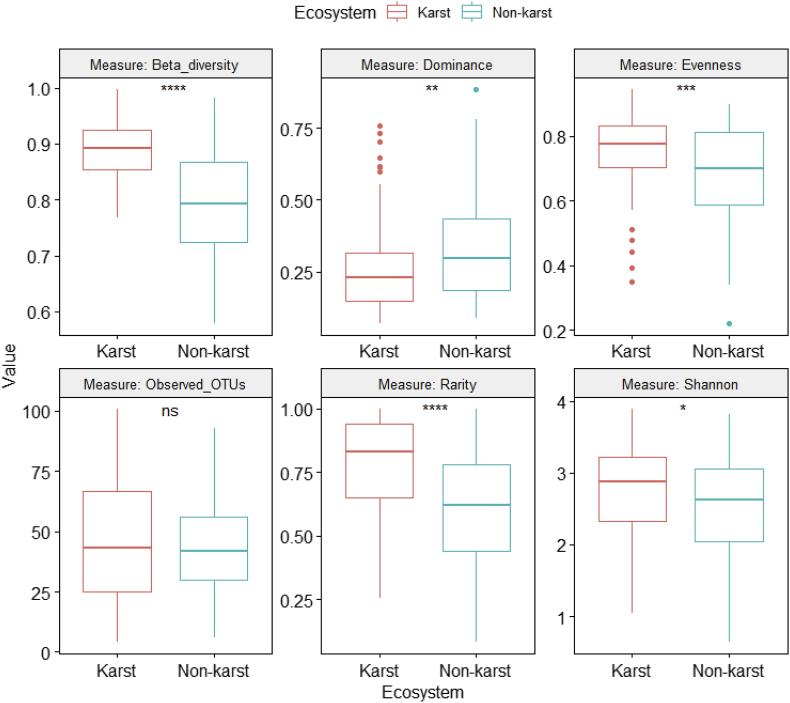
Differences in fungal alpha and beta diversity indices between karst and non-karst soils for the Eurotiomycetes dataset (Supplemental file 7).

**Fig. 7:**
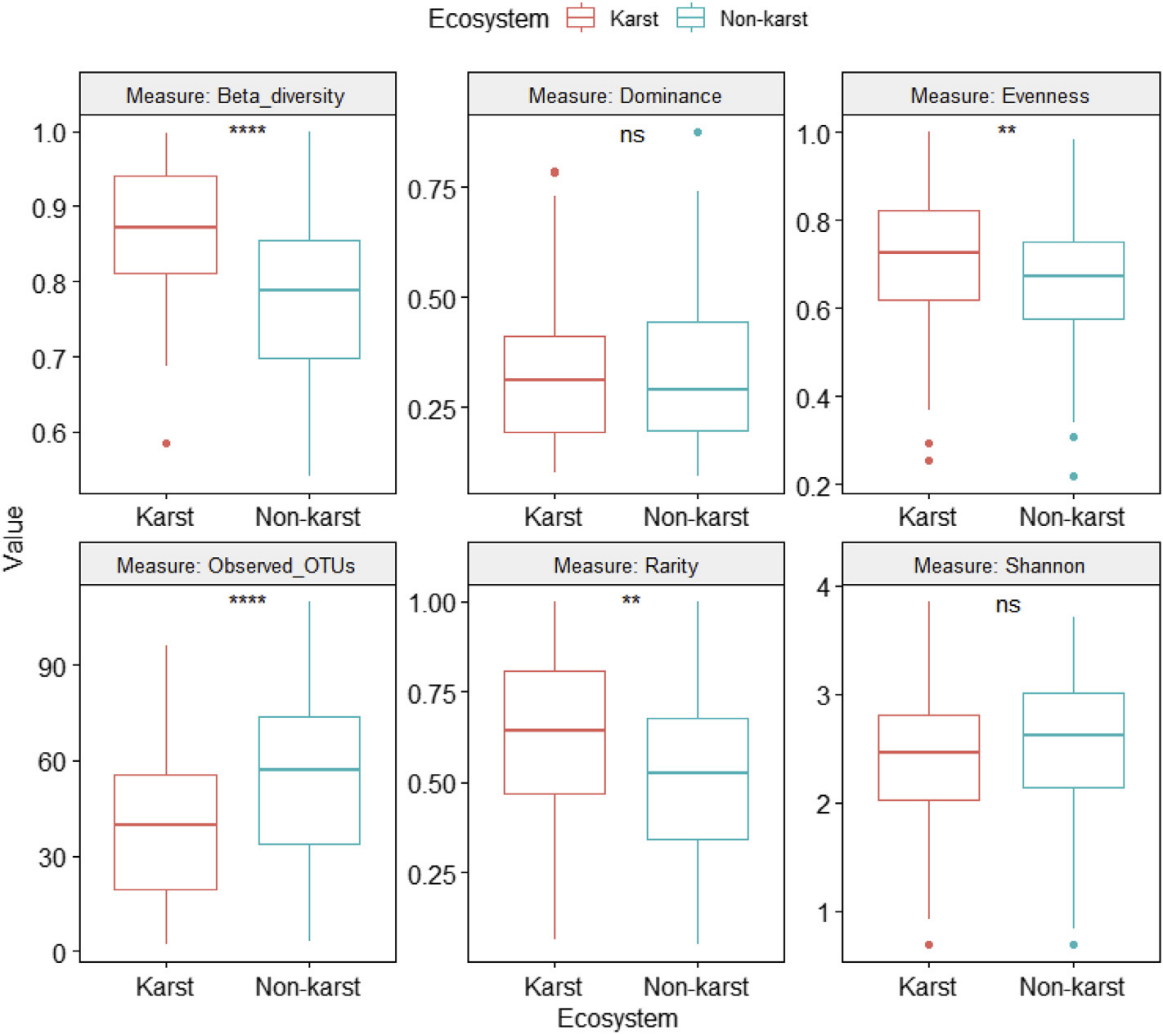
Differences in fungal alpha and beta diversity indices between karst and non-karst soils for the Leotiomycetes dataset (Supplemental file 8).

**Fig. 8:**
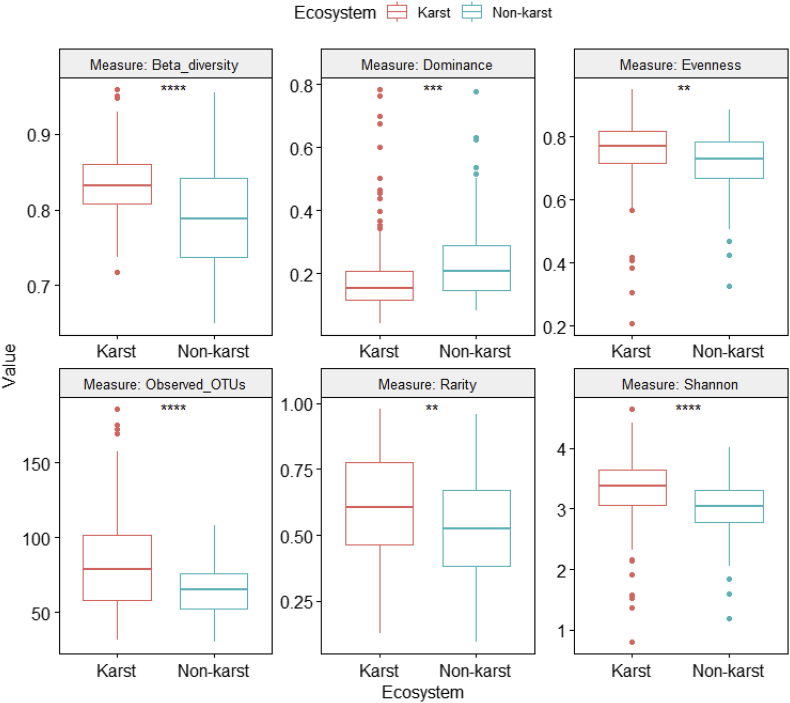
Differences in fungal alpha and beta diversity indices between karst and non-karst soils for the Sordariomycetes dataset (Supplemental file 9).

**Fig. 9:**
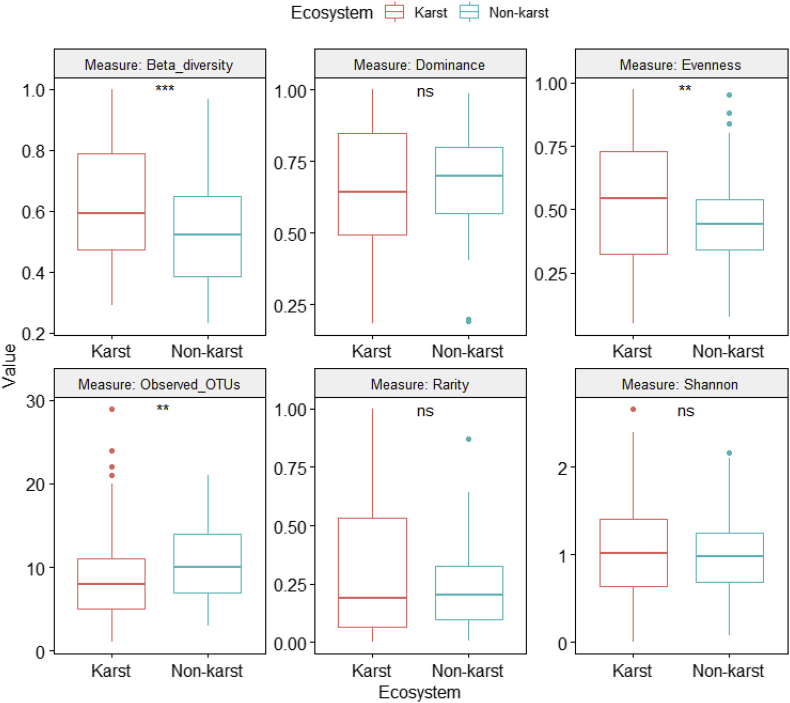
Differences in fungal alpha and beta diversity indices between karst and non-karst soils for the Tremellomycetes dataset (Supplemental file 10).

**Fig. 10:**
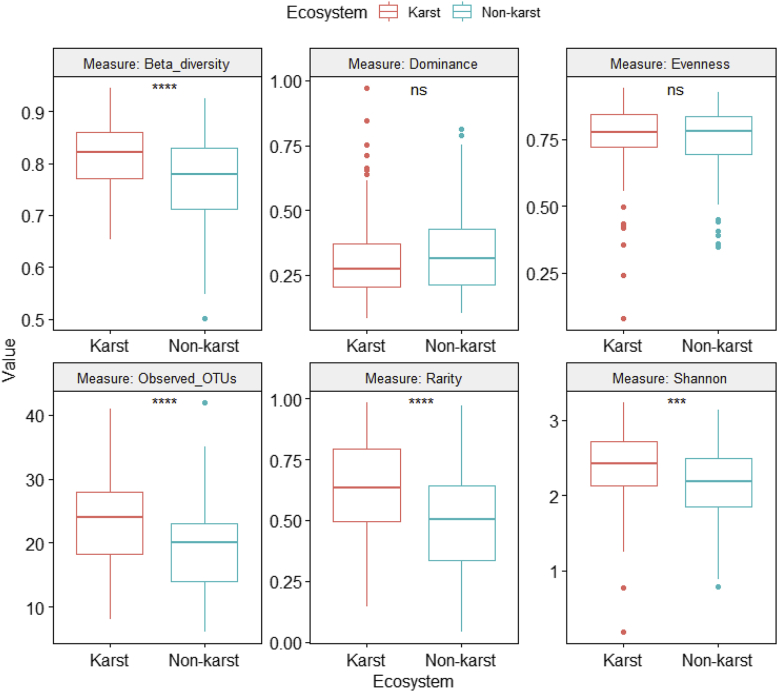
Differences in fungal alpha and beta diversity indices between karst and non-karst soils for the pathotrophs dataset (Supplemental file 11).

**Fig. 11:**
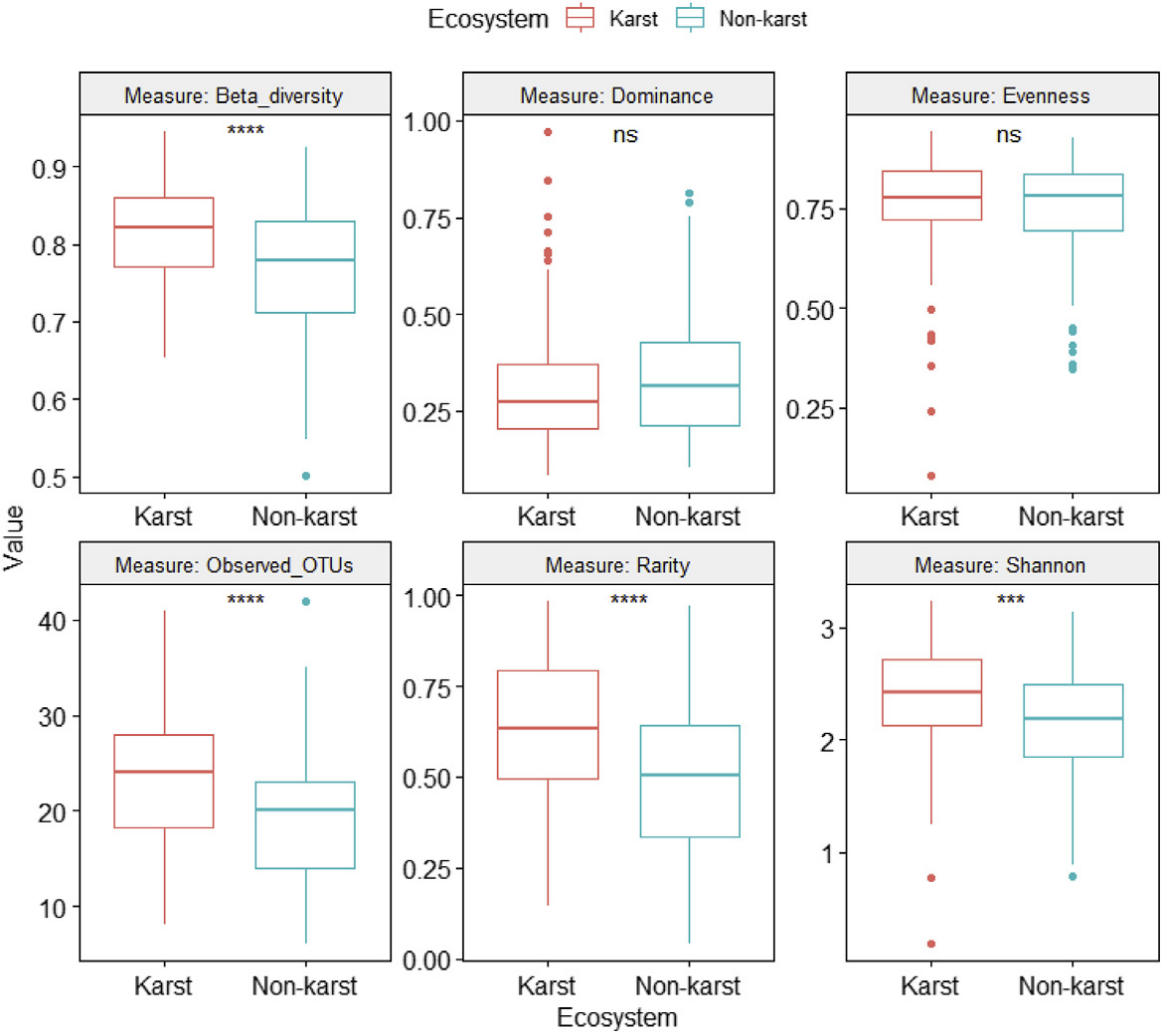
Differences in fungal alpha and beta diversity indices between karst and non-karst soils for the saprotrophs dataset (Supplemental file 12).

**Fig. 12:**
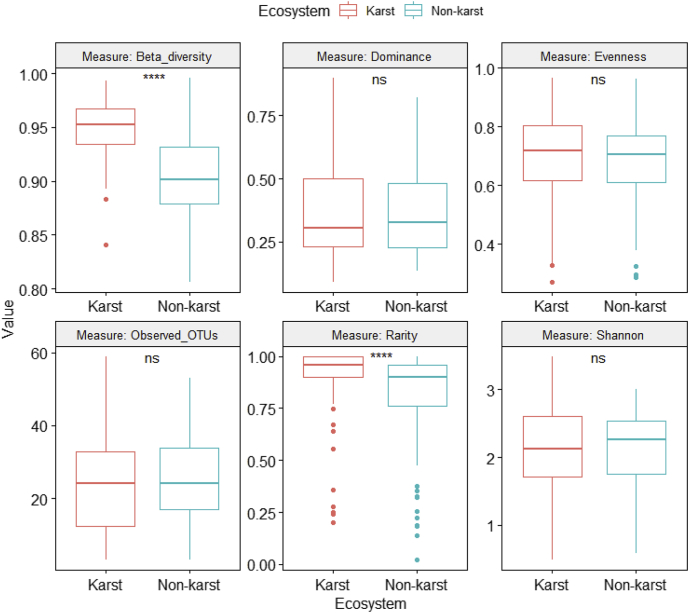
Differences in fungal alpha and beta diversity indices between karst and non-karst soils for the symbiotrophs dataset (Supplemental file 13).

**Fig. 13:**
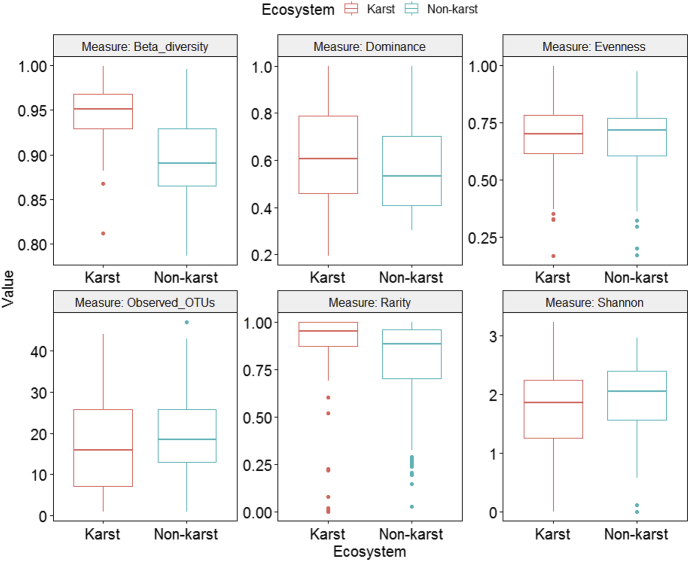
Differences in fungal alpha and beta diversity indices between karst and non-karst soils for the ectomycorrhizal dataset (Supplemental file 14).

### 1.1 Site selection and sampling design

In the present data, Fig. 1 represents rock outcrops, an indicator of whether a particular site was classified as karst (present) or non-karst (absent). Fig. 2 is a schematic representation of how the 15 × 15 m sampling plots were established and how soil samples were collected.

### 1.2 Fungal alpha and beta diversity comparison between karst and non-karst soils

We used One-Way Analysis of Variance (ANOVA) to compare fungal alpha and beta diversity between karst and non-karst soils (Fig. 3, Fig. 4, Fig. 5, Fig. 6, Fig. 7, Fig. 8, Fig. 9, Fig. 10, Fig. 11, Fig. 12, Fig. 13). In Fig. 3, we showed that, for the full dataset (Supplemental file 4), rarefying to equal number of sequences (12,723) per sample (equal sequencing depth) yield qualitatively similar diversity patterns as using the raw data (uneven sequencing depth).

ANOVA was performed on six class-level (Agaricomycetes (Fig. 4, Supplemental file 5), Dothideomycetes (Fig. 5, Supplemental file 6), Eurotiomycetes (Fig. 6, Supplemental file 7), Leotiomycetes (Fig. 7, Supplemental file 8), Sordariomycetes (Fig. 8, Supplemental file 9), Tremellomycetes (Fig. 9, Supplemental file 10)) and four functional-level (pathotrophs (Fig. 10, Supplemental file 11), saprotrophs (Fig. 11, Supplemental file 12), symbiotrophs (Fig. 12, Supplemental file 13), and ectomycorrhizal [EcM] fungi (Fig. 13, Supplemental file 14)) datasets.

## 2 Experimental design, materials, and methods

### 2.1 Study area

This data was collected in Yunnan, southwest China (21.73–25.42 N, 101.41–102.73 E). Yunnan spans approximately 394,000 km^2^ and share boundaries with Guangxi, Guizhou, Sichuan, Tibet, Vietnam, Laos, and Myanmar. The province's topography is characterized by high, rugged mountains, plateaus, and lowlands [6]. Our dataset includes samples from 561 to 2544 m above sea level. Although huge differences in elevation and topography drive large variations in climate, most of Yunnan is subtropical and dominated by seasonal monsoons, with seasonal tropical forests in the southernmost region [6]. Annual precipitation is higher in the southeast and lower in the northwest, and ranges from 600 to 2300 mm, (average 1260 mm). Most of the annual rainfall (∼60%) occur in July and August. Mean temperatures vary between 8 and 17 °C in January and between 11 and 29 °C in July. The northwest is much cooler while the southeast is much warmer than the other regions. The southern region is frost free throughout the year but the northern region may have 210 frost-free days.

### 2.2 Site selection and plot design

We selected 22 karst forests covering a wide range of environmental conditions (elevation, slope, aspect, soil) and 22 non-karst forests at similar altitudes and latitudes to the karst sites. We classified karst and non-karst sites using the presence or absence, respectively, of rock outcrops (Fig. 1). We established five 15 m × 15 m inventory plots in each of the 44 selected karst and non-karst sites (Fig. 2). We placed each plot approximately 50 m from the nearest plot in a crossed transect design (i.e. one in the middle and one each in north, east, west and south directions, Fig. 2). In each plot, we collected soil cores (5 cm in diameter and evenly distributed within the 15 m × 15 m plot) from the topsoil layer (0–20 cm) after removing plant debris and stones, and placed them into sterile zipper bags. We cleaned soil corers using 1:10 bleach solution and rinsed them three times using sterile autoclaved deionized water. We changed nitrile gloves in between plots to avoid sample cross contamination. We homogenized soil samples by mixing thoroughly, transferred representative subsamples into 50 mL sterile Falcon tubes, and stored at −20 °C within 24 hours after collection until DNA isolation.

### 2.3 Environmental factors, soil properties and climate data

We recorded the latitude, longitude and elevation of each plot using a handheld Global Positioning System (GPS, GARMIN GPSMAP 62s). We measured (i) soil pH in a 1:2.5 mixture of soil to water using a digital pH meter (FE28, Mettler Toledo, USA); (ii) total Carbon (TC) and Nitrogen (TN) using a CN analyzer (Vario MAX CN; Elementar, Germany); (iii) total Phosphorus (TP), Potassium (TK), Calcium (TCa) and Magnesium (TMg) using ICP-AES (iCAP6300, Thermo Fisher Scientific, USA). We downloaded the standard 19 bioclimatic variables (BIO1-BIO19) for each sample location at 30 seconds (∼1 km^2^) spatial resolution from Worldclim version 2 [4] using the R raster package [5].

### 2.4 DNA extraction, PCR amplification and sequencing

We homogenized soil samples by vortexing and extracted genomic DNA from 0.5 g representative subsamples using the TIANamp Soil DNA Kit (TIANGEN Biotech Co.,Ltd, Beijing) according to the manufacturer's instructions. We used Qubit 2.0 to quantify DNA concentration and diluted the DNA of each sample to 20 ng/ul final concentration. We amplified the internal transcribed spacer 2 (ITS2) region of the nuclear ribosomal gene using the primers fITS7/ITS4 [2,3] on an Applied Biosystems 2720 Thermal Cycler (Life Technologies, CA, USA). We ran PCR in 25.0 μL reactions using 2.0 μL DNA template, 5.0 μL 5× reaction buffer, 2.0 μL 5 × dNTP (2.5mM), 1.0 μL of each primer (10 μM), 5.0 μL GC buffer, 0.25 μL Q5 DNA polymerase and 8.75 μL ddH2O. We labelled primers with 8 bp barcodes to enable identification of samples after multiplexed sequencing. We applied the following PCR conditions: 95 °C for 2 min followed by 25 cycles at 95 °C for 30 s, 52 °C for 30 s, 72 °C for 30 s and a final elongation at 72 °C for 5 min. We performed PCR reactions in triplicates and pooled the resulting products in equimolar amounts. We extracted PCR products from 2% agarose gels, purified them using the AxyPrep DNA Gel Extraction Kit (Axygen Biosciences, Union City, CA, USA) according to the manufacturer's instructions, and quantified them using Microplate reader (BioTek, FLx800). We prepared sequencing libraries using the TruSeq Nano DNA LT Library Prep Kit, pooled them in equimolar amounts, and paired-end sequenced (2 × 250 bp) them on Illumina Miseq. We deposited the raw sequences at the National Center for Biotechnology Information (NCBI) Sequence Read Archive (SRA) under accession No. SRP158134.

### 2.5 Bioinformatics analysis

We obtained 11,269,934 reads from Illumina Miseq paired-end sequencing. The median length of the forward and reverse raw reads were 244 and 251 bp, respectively. We processed these raw sequences using the freely available bioinformatics software, QIIME 2 version 2018.8 [7]. In brief, we imported demultiplexed forward and reverse reads into QIIME 2 and removed primer/adapter sequences using the cutadapt plugin [7,8]. We then used the DADA2 plugin to denoise (i.e. filter out noisy sequences with expected errors >2.0, correct errors in marginal sequences, remove chimeric sequences, remove singletons, join denoised paired-end reads, and then dereplicate filtered sequences), and cluster (100% OTUs) sequences [9]. We obtained 8,324,409 reads and 39,998 OTUs after this initial quality filtering step. We performed a second quality filtering (uchime-denovo) step to remove chimeras and “borderline chimeras” that were not removed during the first step [10], and obtained 8,272,936 reads and 38,924 OTUs. We performed a final quality control step to filter out OTUs that were present in less than two samples (singletons), resulting in 12,843 OTUs (Supplemental file 1).

### 2.6 Taxonomic and functional guild assignment

We assigned taxonomy to the 12,843 OTUs using a pre-trained Naive Bayes classifier and the q2-feature-classifier plugin [11]. We first trained this classifier using the QIIME release file that corresponds to the Species Hypothesis (SH) from the dynamic use of clustering thresholds of the UNITE fungal ITS database [12]. We successfully assigned 12,831 (out of 12,843) OTUs to the kingdom Fungi, four to Plantae, and eight were unassigned to any Kingdom. We removed all 12 non-fungal OTUs from downstream analyses (Supplemental file 2). We used the FunGuild annotation tool to assign fungal OTUs to ecological guilds (functional groups, Supplemental file 2) based on their taxonomic affinity [13].

### 2.7 Statistical analyses

We estimated the fungal alpha (observed richness, Pielou's evenness, Shannon diversity, dominance, and rarity) and beta diversity using the Microbiome package version 1.9.16 [14]. Beta diversity was quantified as the average dissimilarity of each community from the group mean. Differences in alpha and beta diversity between karst and non-karst fungi were assessed using One-Way Analysis of Variance (ANOVA). We performed these analyses on all fungi combined (Full, Supplemental file 4), on six class-level (Agaricomycetes, Supplemental file 5; Dothideomycetes, Supplemental file 6; Sordariomycetes, Supplemental file 7; Leotiomycetes, Supplemental file 8; Tremellomycetes, Supplemental file 9; and Eurotiomycetes, Supplemental file 10) and on four functional-level (symbiotrophs, Supplemental file 11; pathotrophs, Supplemental file 12; saprotrophs, Supplemental file 13, and EcM fungi, Supplemental file 14) datasets using the R statistical environment [15].
